# Genetic susceptibility to uric acid and selected cardiovascular outcomes: a systematic review and meta-analysis of Mendelian randomization studies

**DOI:** 10.3389/fendo.2026.1880061

**Published:** 2026-07-24

**Authors:** Chunping Li, Ping Chen, Fangda Zheng

**Affiliations:** 1 Department of Cardiology, The Third People’s Hospital of Nanning, Nanning, China; 2 Department of Pulmonary and Critical Care Medicine, The Fourth People’s Hospital of Shenyang of China Medical University, Shenyang, China; 3 Department of Laboratory Medicine, The Fourth People’s Hospital of Shenyang of China Medical University, Shenyang, China

**Keywords:** cardiovascular disease, coronary heart disease, gout, heart failure, hyperuricemia, Mendelian randomization, meta-analysis, serum urate

## Abstract

**Background:**

Serum urate has been associated with cardiovascular disease in observational studies, but whether genetically predicted higher serum urate levels contribute to specific cardiovascular outcomes remains uncertain. We conducted a systematic review and meta-analysis of Mendelian randomization (MR) studies evaluating serum urate-, hyperuricemia-, or gout-related genetic susceptibility in relation to selected cardiovascular outcomes.

**Methods:**

PubMed, Embase, and Web of Science were searched from inception to April 6, 2026. Eligible studies were one-sample or two-sample MR studies assessing relevant urate-related exposures and cardiovascular outcomes. Reporting completeness, overlapping or duplicate estimates, exposure–outcome GWAS sample overlap, and MR-specific risk of bias were assessed before quantitative synthesis. Effect estimates were harmonized and standardized to a 1.5 mg/dL increase in serum urate where applicable. Random-effects inverse-variance meta-analyses, Egger regression tests, leave-one-out analyses, Hartung-Knapp adjusted analyses, and stepwise GWAS-source-restricted sensitivity analyses were performed where applicable.

**Results:**

Thirty-two MR studies reporting 105 MR analyses were included. After estimate-level screening and MR-specific risk-of-bias assessment, 26 MR estimates were retained for quantitative synthesis. In primary analyses, genetically predicted higher serum urate was associated with increased risks of coronary heart disease (OR = 1.13, 95% CI: 1.10–1.15), myocardial infarction (OR = 1.13, 95% CI: 1.08–1.17), coronary artery disease (OR = 1.17, 95% CI: 1.08–1.26), atrial fibrillation (OR = 1.07, 95% CI: 1.02–1.12), and heart failure based on IVW estimates (OR = 1.08, 95% CI: 1.05–1.11). However, the atrial fibrillation association did not remain statistically significant after Hartung-Knapp adjustment and was reduced to a single estimate after GWAS-source restriction. In GWAS-source-restricted sensitivity analyses, only myocardial infarction and coronary artery disease retained at least three estimates and remained positively associated with serum urate.

**Conclusion:**

Evidence was most consistent for myocardial infarction and coronary artery disease. Findings for coronary heart disease, atrial fibrillation, and heart failure were more limited after stricter source restriction. These results do not establish a broad causal effect on cardiovascular disease as a whole or direct cardiovascular benefit from urate-lowering therapy.

**Systematic Review Registration:**

https://www.crd.york.ac.uk/PROSPERO/, identifier CRD420261380161

## 1 Introduction

Cardiovascular disease remains a leading contributor to global disease burden and mortality, with ischemic heart disease and stroke consistently ranking among the major causes of death and disability. Although established risk factors such as hypertension, dyslipidemia, impaired glucose metabolism, smoking, and obesity have been well characterized, substantial residual cardiovascular risk persists, underscoring the need to identify additional modifiable metabolic factors. Serum urate, the end product of purine metabolism, has attracted increasing attention because of its close association with cardiovascular and metabolic abnormalities. The Global Burden of Cardiovascular Diseases study showed that ischemic heart disease accounted for the highest age-standardized disability-adjusted life-year burden among all diseases, highlighting the public health importance of identifying potential risk factors (1).

Elevated serum urate is not only the central biological substrate for gout but may also participate in cardiovascular pathophysiology. Experimental and clinical evidence suggests that urate may contribute to hypertension, atherosclerosis, and cardiovascular events through endothelial dysfunction, oxidative stress, inflammation, activation of the renin-angiotensin system, vascular smooth muscle cell proliferation, and renal microvascular injury (2, 3). However, urate also has antioxidant properties, and its cardiovascular effects may depend on disease context, renal function, metabolic status, and medication use. Therefore, the role of urate in cardiovascular disease remains controversial (2, 3).

Previous observational studies and systematic reviews have generally reported associations of higher serum urate levels or hyperuricemia with coronary heart disease, stroke, heart failure, hypertension, and all-cause mortality. However, observational associations cannot fully account for residual confounding or reverse causation. For example, impaired renal function, obesity, insulin resistance, diuretic use, and dietary factors may influence both serum urate levels and cardiovascular risk, making it difficult for conventional epidemiological studies to determine whether elevated serum urate is an independent causal factor for cardiovascular disease. An umbrella review incorporating observational studies, randomized controlled trials, and Mendelian randomization studies also indicated that, although serum urate is associated with multiple health outcomes, the strength of evidence varies across study designs (4).

Mendelian randomization (MR) uses genetic variants associated with an exposure as instrumental variables and can reduce residual confounding and reverse causation in conventional observational studies. It has therefore been widely applied to evaluate potential causal relationships between modifiable risk factors and disease outcomes (5, 6). With the development of publicly available genome-wide association study (GWAS) summary data and two-sample MR platforms, researchers can systematically integrate genetic associations for exposures and outcomes to assess the potential causal effects of genetically predicted increases in serum urate levels on cardiovascular disease risk (7).

However, existing MR studies have reported inconsistent findings regarding the causal relationship between genetically predicted increases in serum urate levels and cardiovascular disease. Some studies did not support a clear causal association of genetically predicted higher serum urate levels with coronary heart disease, ischemic stroke, or heart failure (8). In contrast, studies using urate-related genetic markers have suggested that genetic predisposition to hyperuricemia may be associated with increased cardiovascular event risk in high-risk populations (9), and other evidence has suggested that urate may be related to cardiovascular events (10). These discrepancies may be attributable to differences in ancestry, genetic instrument selection, exposure and outcome GWAS sources, MR estimation methods, and strategies for controlling pleiotropy. Therefore, findings from individual MR studies require further evaluation through systematic evidence synthesis and stratified analyses.

Accordingly, there is a need to systematically integrate existing MR evidence to clarify the relationship between genetically predicted increases in serum urate levels and specific cardiovascular disease subtypes. We therefore conducted a systematic review and meta-analysis of MR studies evaluating genetic susceptibility related to serum urate, hyperuricemia, or gout in relation to cardiovascular outcomes. We focused primarily on genetically predicted increases in serum urate levels and major atherosclerotic cardiovascular outcomes.

## 2 Materials and methods

### 2.1 Data sources and search strategy

The protocol for this systematic review and meta-analysis of MR studies was prospectively registered in the International Prospective Register of Systematic Reviews (PROSPERO; CRD420261380161) and followed the Preferred Reporting Items for Systematic Reviews and Meta-Analyses (PRISMA) guideline.

**Figure 1:**
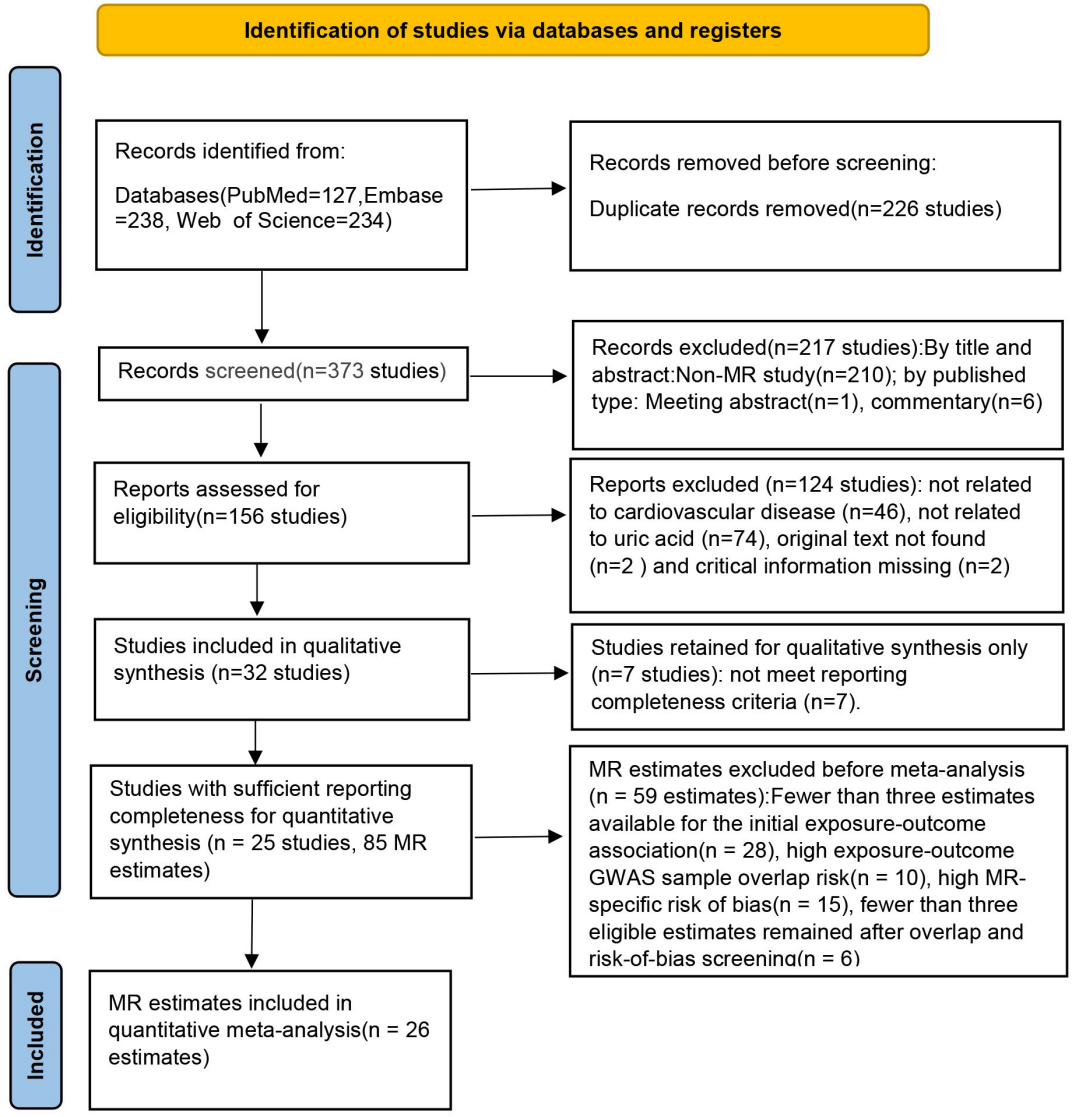
PRISMA flowchart of study selection process.

A comprehensive literature search was conducted in PubMed, EMBASE, and Web of Science from database inception to April 6, 2026. The study selection process is summarized in the flow diagram (**Figure 1**). The complete search strategies for each database are provided in **Supplementary Table 1**.

### 2.2 Study selection

Retrieved records were imported into EndNote 2025 for duplicate identification and removal. Two reviewers (C.L. and P.C.) independently assessed the remaining records and selected studies according to predefined eligibility criteria. Disagreements were resolved by consultation with a third investigator (F.Z.).

Eligible studies were unidirectional or bidirectional MR studies using either one-sample or two-sample designs that assessed associations between genetic variants related to serum urate, hyperuricemia, or gout and any cardiovascular disease outcome. Primary outcomes of interest included angina, myocardial infarction, coronary heart disease and coronary artery disease. Secondary cardiovascular outcomes included stroke, systolic blood pressure, diastolic blood pressure, hypertension, heart failure, and atrial fibrillation.

Studies were excluded if they met any of the following criteria: (1) non-genetic studies or studies not using MR analysis; (2) animal studies; (3) pediatric studies with participants younger than 18 years; (4) case reports, narrative reviews, or other non-original research articles.

### 2.3 Data extraction and reporting completeness assessment

From each eligible MR study, we extracted the first author’s surname, publication year, ancestry of the study population, exposure definition, outcome definition, MR design, MR method, participating genomic consortia or data sources, sample size, genetic instrument information, effect estimates, and corresponding 95% confidence intervals.

We first assessed reporting completeness using a 20-item checklist adapted from STROBE-MR and tailored to the characteristics of MR studies. This assessment was used to determine whether the original report provided sufficient methodological and numerical information for further quantitative synthesis, and was not treated as a risk-of-bias assessment. Two independent reviewers applied a three-level scoring system: 2 points for complete reporting, 1 point for partial reporting, and 0 points for no reporting. Total scores were converted into percentages and classified as high reporting completeness (>85%), moderate reporting completeness (75%–85%), or low reporting completeness (<75%). The >85% threshold corresponded to at least 35 of 40 possible points and was used as a pragmatic threshold to ensure that sufficient methodological and numerical information was available for estimate-level eligibility assessment, harmonization, sample-overlap evaluation, and risk-of-bias judgement. This threshold was not intended to classify study quality or risk of bias. Studies that did not meet the high reporting completeness threshold were retained for qualitative synthesis only and were not carried forward to estimate-level quantitative assessment. The 20-item scoring criteria for reporting completeness are provided in **Supplementary Table 2**.

### 2.4 Estimate-level selection for quantitative synthesis and MR-specific risk-of-bias assessment

Quantitative synthesis was conducted at the MR estimate level rather than only at the study level. Among studies with sufficient reporting completeness, all potentially eligible MR estimates were assessed for comparability, potential data-source independence, exposure–outcome GWAS sample overlap, and MR-specific risk of bias before meta-analysis.

For estimate-level selection, estimates were considered eligible for pooling only when they assessed comparable exposure and outcome definitions, used compatible effect scales after harmonization, and belonged to a planned meta-analysis group with at least three eligible estimates. When multiple estimates were available for the same planned meta-analysis group and were based on overlapping or duplicate data sources, only one estimate was retained. Preference was given to the estimate with the larger sample size, more independent exposure and outcome data sources, lower risk of sample overlap, or more complete methodological reporting.

We then assessed potential sample overlap between exposure and outcome GWAS sources. Estimates with high exposure–outcome GWAS sample-overlap risk were excluded from quantitative synthesis. Estimates with possible or unclear overlap were not automatically excluded, but this uncertainty was incorporated into the sample-overlap domain of the MR-specific risk-of-bias assessment.

After assessment of potential exposure–outcome GWAS sample overlap and before the MR-specific risk-of-bias assessment, estimates originating from the same original publication but representing different exposure–outcome comparisons or different exposure or outcome GWAS sources were sequentially labelled using suffixes, such as a–z. This labelling was used to ensure traceability of estimate-level data sources and to distinguish suffix-labelled estimates from simple duplicate estimates. Suffix labelling itself was not considered evidence of statistical independence; potential residual dependence related to shared original publications or shared or closely related GWAS sources was further examined in the stepwise GWAS-source-restricted sensitivity analyses.

MR-specific risk of bias was assessed using a domain-based tool developed for this review (**Supplementary Table 3**). The domains included weak instrument bias, genetic confounding bias, bias from inappropriate covariate adjustment, horizontal pleiotropy bias, population stratification bias, sample overlap bias, winner’s curse bias, and directionality or reverse causation bias. Each domain was judged as low, moderate, or high risk. The weighting scheme was prespecified to give greater emphasis to domains most directly related to the core MR assumptions and causal interpretation, including instrument strength, horizontal pleiotropy, sample overlap, and directionality. Core MR domains, including weak instrument bias, horizontal pleiotropy bias, sample overlap bias, and directionality or reverse causation bias, were assigned 4, 2, and 0 points for low, moderate, and high risk, respectively; the remaining domains were assigned 2, 1, and 0 points. The maximum score was 24. Any high-risk domain resulted in an overall high-risk judgement. Among estimates without any high-risk domain, scores of 22 or higher indicated low risk, scores of 16 to 21 indicated moderate risk, and scores of 15 or lower indicated high risk. Estimates with high overall MR-specific risk of bias were excluded from quantitative synthesis.

### 2.5 Statistical analysis

Meta-analyses were conducted according to the eligibility rules defined above. Quantitative synthesis was restricted to the primary IVW-based estimates reported by the original studies. When both IVW and IVW-RE estimates were available and represented distinct primary analyses, they were not combined in the same meta-analysis. Other MR sensitivity methods, such as weighted median, MR-Egger, or MR-PRESSO, were not pooled in the meta-analysis.

Because definitions and data sources for coronary heart disease (CHD) and coronary artery disease (CAD) differed across original MR studies, we retained the outcome labels reported in the original studies and analyzed CHD and CAD separately.

All effect estimates were harmonized and standardized. For binary outcomes, original odds ratios (ORs) and corresponding 95% CIs were directly extracted. For continuous outcomes, when permitted by the original study reports, effect estimates were converted to OR-equivalent estimates to allow analysis on a common scale. To account for differences in the standard deviation (SD) of serum urate across studies, all effect estimates were standardized to a target SD of 1.5 mg/dL using the following formula:


$$OR_{standardized}=exp\left[\log(OR_{original})\times \frac{1.5}{SD_{study-specific}}\right]$$

where 1.5 mg/dL was the target SD and SDstudy−specific represented the serum urate SD reported in each original study. The lower and upper confidence limits were standardized using the same transformation.

Based on the standardized ORs and corresponding 95% CIs, effect estimates were transformed to the log(OR) scale, and the corresponding SE(log(OR)) was calculated. Meta-analyses were performed on the log(OR) scale using inverse-variance weighting, and pooled estimates were exponentiated and reported as ORs with 95% CIs. Pooled analyses were conducted using the metagen function in the meta package, version 8.2.0. Between-study heterogeneity was assessed using Cochran’s Q test, I2 statistic, and τ2. An I2 value >75% together with a Q-test P value<0.05 was considered indicative of substantial heterogeneity. Given the potential heterogeneity across MR studies in ancestry, genetic instrument selection, and exposure and outcome GWAS data sources, a random-effects model with τ2 estimated by restricted maximum likelihood (REML) was used as the primary analytical model.

Publication bias and small-study effects were assessed using funnel plots and Egger regression tests when at least three estimates were available. For the Egger regression test, P< 0.05 was considered suggestive of funnel plot asymmetry or potential small-study effects. Because several outcomes included a limited number of estimates, Egger regression tests were interpreted as exploratory screening for funnel plot asymmetry rather than definitive evidence for or against publication bias or small-study effects. When the Egger test indicated funnel plot asymmetry, the trim-and-fill method was further applied to evaluate the potential influence of asymmetry on pooled estimates.

Leave-one-out sensitivity analyses were conducted by omitting one estimate at a time to assess whether pooled results were driven by a single estimate. Hartung-Knapp adjusted random-effects analyses were additionally performed for eligible meta-analysis groups to evaluate the influence of small numbers of estimates on interval estimation and statistical inference. These analyses used the same random-effects framework and between-study variance estimator as the primary model, but applied Hartung-Knapp adjustment to the confidence intervals and statistical inference. Fixed-effect models were not used as routine sensitivity analyses because a common underlying causal effect across MR studies was not assumed.

To further address residual dependence among pooled estimates arising from shared exposure or outcome GWAS sources, stepwise GWAS-source-restricted sensitivity analyses were performed for each outcome- and method-specific meta-analysis group. Exposure and outcome GWAS sources were grouped according to the named GWAS resource, consortium, or substantially overlapping dataset. Publication identity alone was not used as a rigid exclusion criterion, because suffix-labelled estimates from the same MR article could represent different exposure GWAS sources, outcome GWAS datasets, ancestry groups, or cardiovascular phenotype definitions.

A stepwise exposure-first approach was used. First, among estimates sharing the same exposure GWAS source within an outcome-specific meta-analysis group, only one estimate was retained. Second, among the remaining estimates, only one estimate was retained for each outcome GWAS source. When more than one estimate was eligible within the same or closely related GWAS source, the retained estimate was selected according to a prespecified hierarchy: lower overall MR-specific risk of bias, lower exposure–outcome sample-overlap risk, greater independence between exposure and outcome GWAS sources, larger exposure or outcome sample size, clearer outcome definition, and more complete methodological reporting. Statistical significance, effect size, or direction of association was not used as a selection criterion.

Restricted meta-analysis was performed only when at least three GWAS-source-restricted estimates remained. These restricted analyses used the same log(OR)-scale random-effects inverse-variance model and REML estimator as the primary analysis. When fewer than three estimates remained after GWAS-source restriction, meta-analysis was not performed and the evidence was summarized descriptively.

To explore between-study heterogeneity potentially attributable to ancestry, ancestry subgroup analyses were conducted when at least three eligible estimates were available within a subgroup. Subgroups with fewer than three estimates were not pooled and were presented descriptively only. Formal between-ancestry comparisons were not interpreted when any subgroup contained fewer than three estimates. Because the number of estimates for each outcome was limited, multivariable meta-regression was not performed.

All analyses were performed using R, version 4.4.1. All statistical tests were two-sided, with P< 0.05 considered statistically significant. Pooled effects were expressed as ORs with 95% CIs; an effect was considered statistically significant when the 95% CI did not include the null value of 1.

## 3 Results

### 3.1 Study selection and included studies

Using the predefined search strategy, 599 records were identified. After duplicate removal, title and abstract screening, and full-text assessment, 32 MR studies were included (11–42). The study selection process is shown in **Figure 1**.

Among the 32 included studies, 7 did not meet the reporting completeness threshold for quantitative assessment and were retained for qualitative synthesis only (**Supplementary Table 4**). The remaining 25 studies provided 85 MR estimates for estimate-level quantitative assessment. After handling overlapping or duplicate estimates, screening for exposure-outcome GWAS sample overlap, and applying the MR-specific risk-of-bias assessment, 26 MR estimates were retained for the final quantitative meta-analysis. The overall estimate-level selection process is summarized in **Figure 1**.

### 3.2 Systematic review findings

#### 3.2.1 Study characteristics

The 32 included studies reported 105 MR analyses, of which 27 used a two-sample MR design and 5 used a one-sample MR design. Sample sizes ranged from 8,332 to 1,843,063 participants, and the number of SNPs used as genetic instruments ranged from 2 to 314. Among these analyses, 81 had ancestry-matched exposure and outcome data, including 75 analyses in European ancestry populations and 6 in Asian ancestry populations; the remaining 24 analyses involved mixed-ancestry populations. Except for one study (41), most studies conducted sensitivity analyses to assess the robustness of their primary MR findings.

Among all MR analyses, 98 evaluated associations between genetically predicted increases in serum urate levels and cardiovascular outcomes. These analyses covered a broad range of cardiovascular traits and disease subtypes, including angina, coronary heart disease, myocardial infarction, coronary artery disease, overall cardiovascular disease, stroke and stroke subtypes, atrial fibrillation, heart failure, hypertension, systolic blood pressure, diastolic blood pressure, and peripheral artery disease. Specifically, the serum urate analyses included angina (n = 6), coronary heart disease (n = 15), myocardial infarction (n = 12), coronary artery disease (n = 11), cardiovascular disease (n = 1), stroke (n = 8), ischemic stroke (n = 5), cardioembolic stroke (n = 2), intracerebral hemorrhage (n = 2), large artery stroke (n = 2), small vessel stroke (n = 2), atrial fibrillation (n = 10), heart failure (n = 10), hypertension (n = 5), systolic blood pressure (n = 4), diastolic blood pressure (n = 2), and peripheral artery disease (n = 1). In addition, 2 analyses evaluated genetically predicted hyperuricemia in relation to cardiovascular disease, and 5 analyses evaluated genetically predicted gout in relation to cardiovascular outcomes, including cardiovascular disease (n = 2), angina (n = 1), hypertension (n = 1), and atrial fibrillation (n = 1).

#### 3.2.2 Reporting completeness, sample-overlap screening, and MR-specific risk-of-bias assessment

The reporting completeness assessment identified 25 studies with high reporting completeness, which were carried forward to estimate-level quantitative assessment; the remaining 7 studies were retained for qualitative synthesis only (**Supplementary Table 4**).

After the study-level reporting completeness assessment, estimates from studies with high reporting completeness were evaluated at the estimate level. Planned meta-analysis groups with fewer than three MR estimates after reporting completeness assessment were not carried forward for quantitative synthesis (**Supplementary Table 5**). For groups with sufficient estimates, overlapping or duplicate MR estimates within the same planned meta-analysis group were identified and handled before pooling (**Supplementary Table 6**). The exposure and outcome characteristics of MR estimates carried forward to MR-specific risk-of-bias assessment are summarized in **Supplementary Table 7**. Major exposure and outcome GWAS sources used by the retained MR estimates, including serum urate GWAS resources and cardiovascular outcome GWAS datasets, were also extracted and documented in **Supplementary Table 7** (43–49).

Potential sample overlap between exposure and outcome GWAS sources was then evaluated (**Supplementary Table 8**). Estimates with high exposure–outcome GWAS sample-overlap risk were excluded, whereas possible or unclear overlap was incorporated into the sample-overlap domain of the subsequent MR-specific risk-of-bias assessment. Estimates with high overall MR-specific risk of bias were excluded from quantitative synthesis. Exposure–outcome associations with fewer than three eligible MR estimates remaining after MR-specific risk-of-bias assessment were not pooled (**Supplementary Tables 9**, **10**).

### 3.3 Meta-analysis of coronary outcomes

After estimate-level screening, quantitative meta-analysis was performed for coronary heart disease, myocardial infarction, and coronary artery disease.

**Figure 2:**
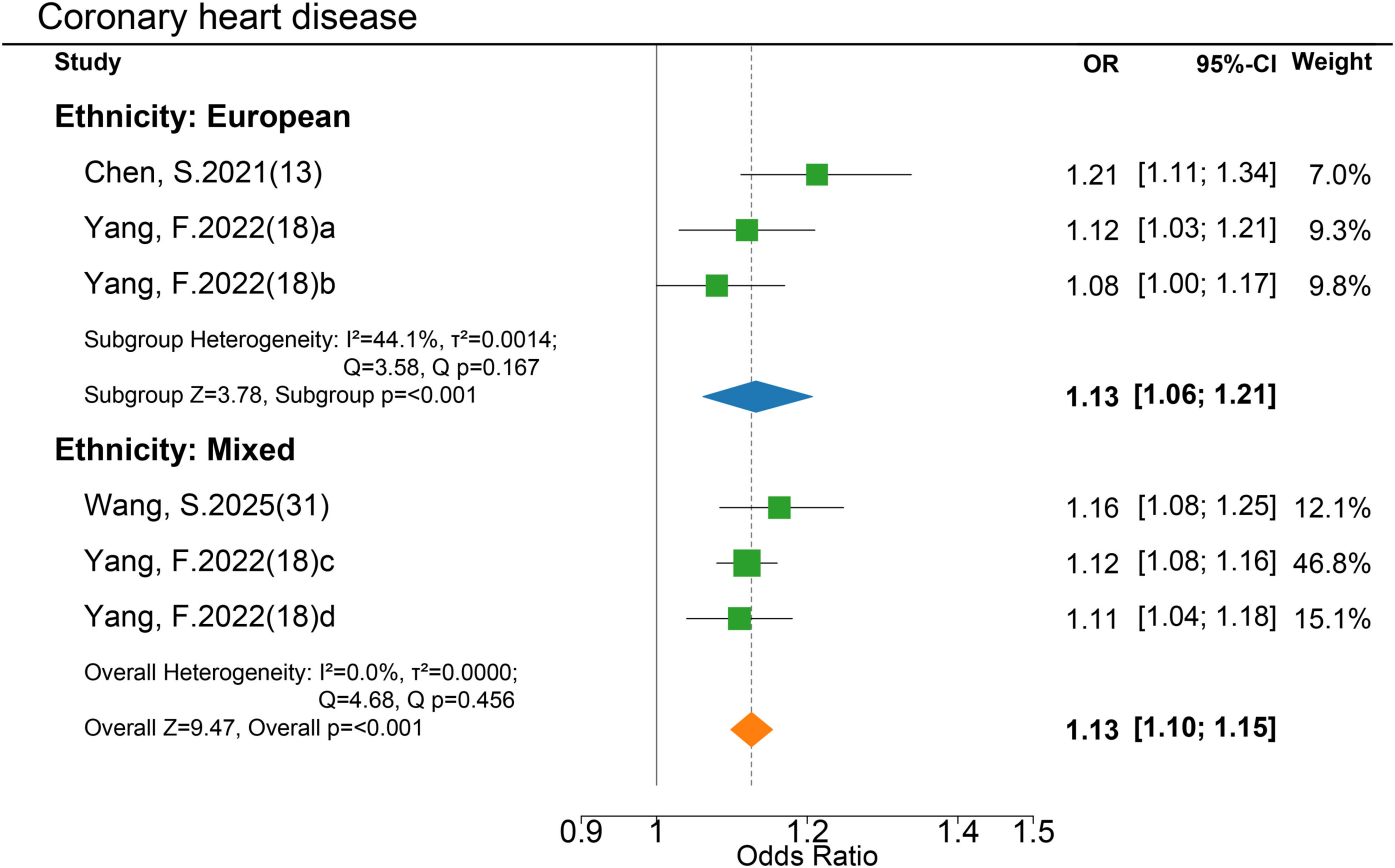
Meta-analysis of genetically predicted serum urate and coronary heart disease. Forest plot showing the association between genetically predicted higher serum urate levels and coronary heart disease. Effect estimates are presented as odds ratios (ORs) with 95% confidence intervals (CIs), standardized to a 1.5 mg/dL increase in serum urate where applicable. Individual study estimates are shown as green squares, with square size proportional to the study weight; horizontal lines indicate 95% CIs. The blue diamond represents the pooled estimate for the European ancestry subgroup, and the orange diamond represents the overall pooled estimate. Estimates were synthesized using a random-effects inverse-variance model. CHD, coronary heart disease; OR, odds ratio; CI, confidence interval; I^2^, inconsistency statistic; τ^2^, between-study variance; Q, Cochran’s Q statistic.

For coronary heart disease, 6 MR estimates retained after estimate-level screening were included. Genetically predicted higher serum urate was associated with a higher risk of coronary heart disease (OR = 1.13, 95% CI: 1.10–1.15; I^2^ = 0.0%; τ^2^ = 0.0000; Q = 4.68, P = 0.456; **Figure 2**). In the European ancestry subgroup, the association was directionally consistent and statistically significant (OR = 1.13, 95% CI: 1.06–1.21; I^2^ = 44.1%; τ^2^ = 0.0014; Q = 3.58, P = 0.167).

**Figure 3:**
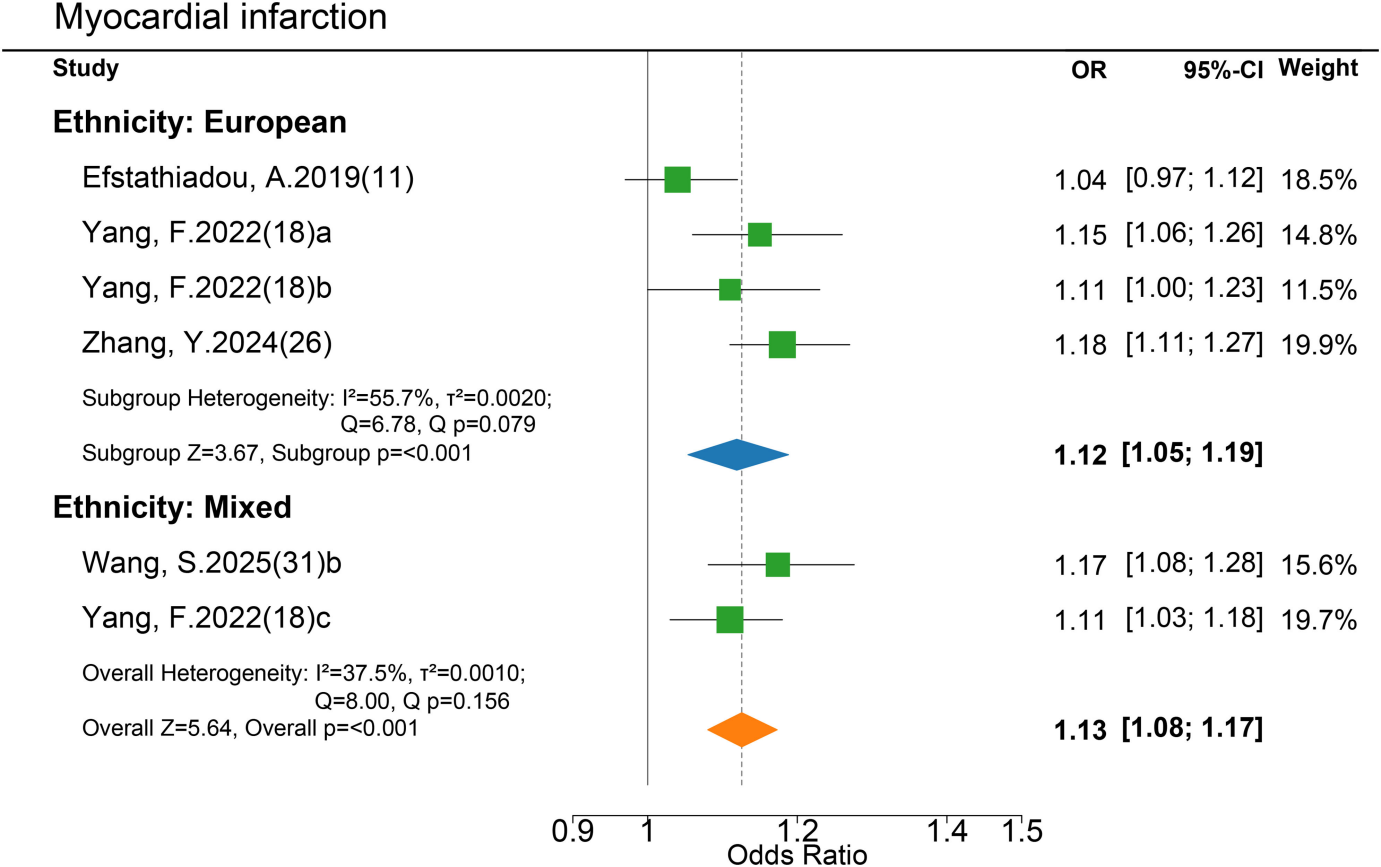
Meta-analysis of genetically predicted serum urate and myocardial infarction. Forest plot showing the association between genetically predicted higher serum urate levels and myocardial infarction. Effect estimates are presented as odds ratios (ORs) with 95% confidence intervals (CIs), standardized to a 1.5 mg/dL increase in serum urate where applicable. Individual study estimates are shown as green squares, with square size proportional to the study weight; horizontal lines indicate 95% CIs. The blue diamond represents the pooled estimate for the European ancestry subgroup, and the orange diamond represents the overall pooled estimate. Estimates were synthesized using a random-effects inverse-variance model. MI, myocardial infarction; OR, odds ratio; CI, confidence interval; I^2^, inconsistency statistic; τ^2^, between-study variance; Q, Cochran’s Q statistic.

For myocardial infarction, 6 MR estimates retained after estimate-level screening were included. The pooled analysis showed a positive association between genetically predicted higher serum urate and myocardial infarction risk (OR = 1.13, 95% CI: 1.08–1.17; I^2^ = 37.5%; τ^2^ = 0.0010; Q = 8.00, P = 0.156; **Figure 3**). The European ancestry subgroup showed a similar positive association (OR = 1.12, 95% CI: 1.05–1.19; I^2^ = 55.7%; τ^2^ = 0.0020; Q = 6.78, P = 0.079).

**Figure 4:**
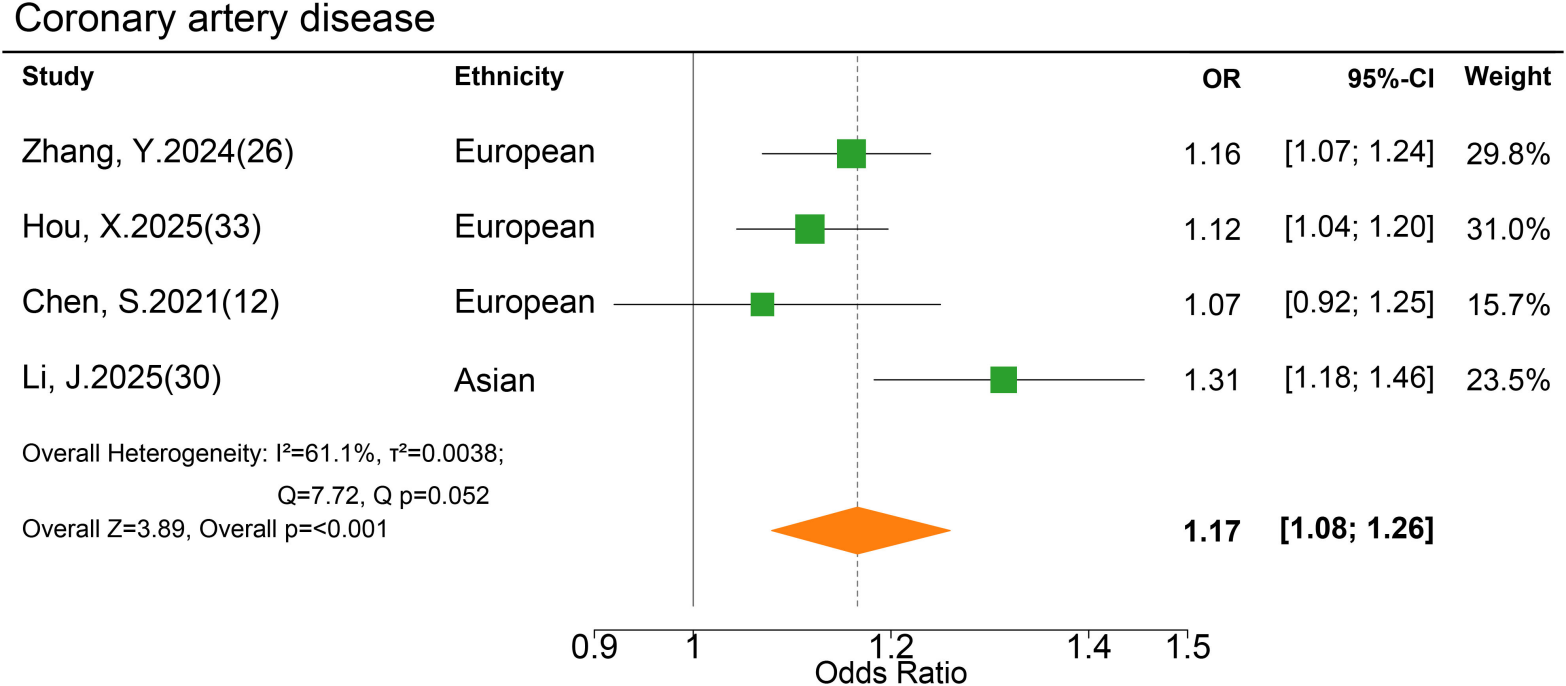
Meta-analysis of genetically predicted serum urate and coronary artery disease. Forest plot showing the overall association between genetically predicted higher serum urate levels and coronary artery disease. Effect estimates are presented as odds ratios (ORs) with 95% confidence intervals (CIs), standardized to a 1.5 mg/dL increase in serum urate where applicable. Individual study estimates are shown as green squares, with square size proportional to the random-effects weight; horizontal lines indicate 95% CIs. The orange diamond represents the overall pooled estimate. Ancestry information is displayed descriptively only, and no ancestry-specific subgroup pooling is shown in this figure. The Asian ancestry estimate was included in the overall meta-analysis but was not pooled as a separate ancestry subgroup because only one eligible Asian estimate remained. The European ancestry-restricted analysis is presented separately in **Supplementary Figure 1**. Estimates were synthesized using a random-effects inverse-variance model. CAD, coronary artery disease; OR, odds ratio; CI, confidence interval; I^2^, inconsistency statistic; τ^2^, between-study variance; Q, Cochran’s Q statistic.

For coronary artery disease, 4 MR estimates retained after estimate-level screening were included. Genetically predicted higher serum urate was associated with a higher risk of coronary artery disease (OR = 1.17, 95% CI: 1.08–1.26; I^2^ = 61.1%; τ^2^ = 0.0038; Q = 7.72, P = 0.052; **Figure 4**). In the European ancestry subgroup, the pooled estimate remained positive, with no observed heterogeneity (OR = 1.13, 95% CI: 1.08–1.19; I^2^ = 0.0%; τ^2^ = 0.0000; Q = 1.07, P = 0.587; **Supplementary Figure 1**). One Asian ancestry estimate was retained in the overall analysis and showed a positive association individually, but it was not pooled as a separate ancestry subgroup because only one eligible Asian estimate remained. Because CHD, MI, and CAD were related but non-identical coronary phenotypes and used different outcome GWAS definitions across original MR studies, these outcomes were analyzed and interpreted separately.

### 3.4 Meta-analysis of atrial fibrillation and heart failure

**Figure 5:**
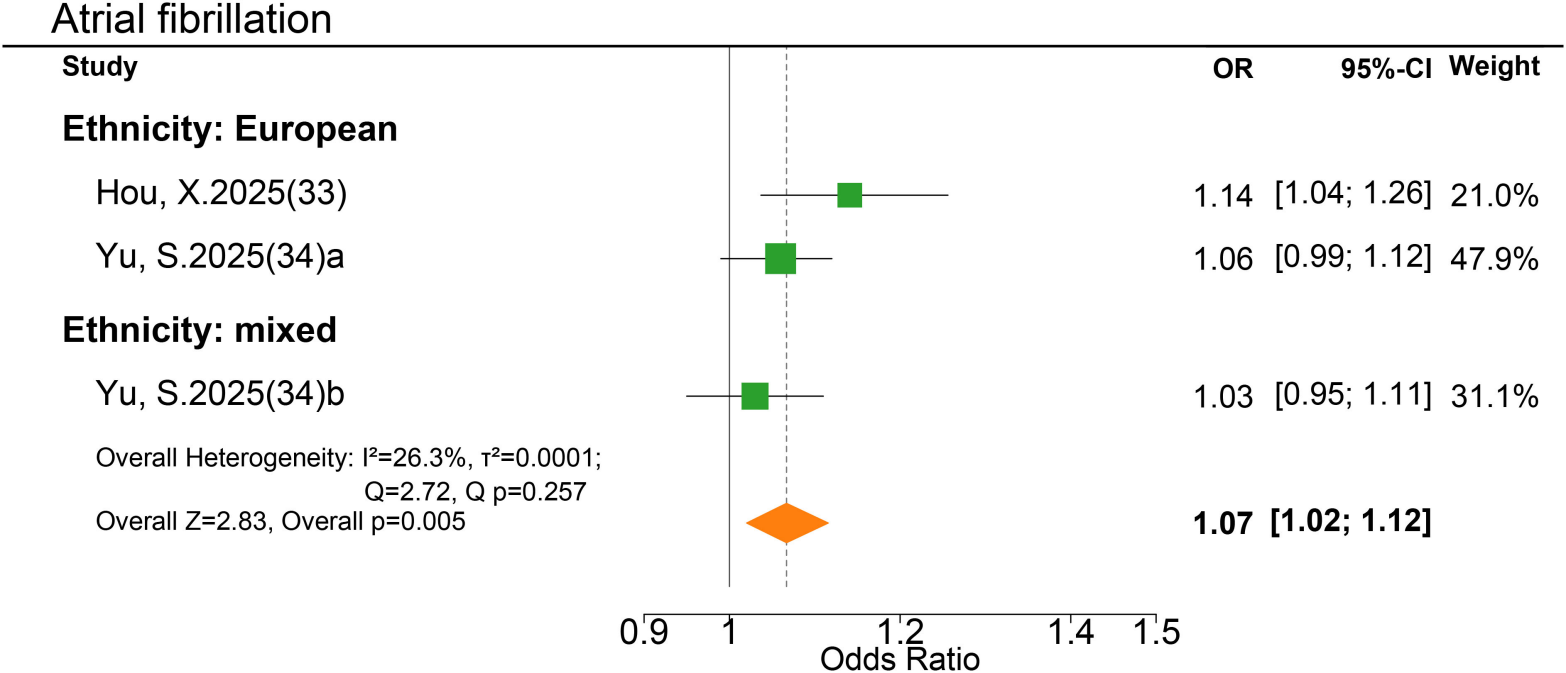
Meta-analysis of genetically predicted serum urate and atrial fibrillation. Forest plot showing the association between genetically predicted higher serum urate levels and atrial fibrillation. Effect estimates are presented as odds ratios (ORs) with 95% confidence intervals (CIs), standardized to a 1.5 mg/dL increase in serum urate where applicable. Individual study estimates are shown as green squares, with square size proportional to the study weight; horizontal lines indicate 95% CIs. The orange diamond represents the overall pooled estimate. Ancestry-specific findings were considered descriptive because fewer than three eligible estimates were available within at least one ancestry subgroup, and formal between-ancestry comparison was not performed. Estimates were synthesized using a random-effects inverse-variance model. AF, atrial fibrillation; OR, odds ratio; CI, confidence interval; I^2^, inconsistency statistic; τ^2^, between-study variance; Q, Cochran’s Q statistic.

For atrial fibrillation, 3 MR estimates retained after estimate-level screening were included. The pooled analysis suggested a positive association between genetically predicted higher serum urate and atrial fibrillation risk (OR = 1.07, 95% CI: 1.02–1.12; I^2^ = 26.3%; τ^2^ = 0.0001; Q = 2.72, P = 0.257; **Figure 5**). However, because this analysis was based on only three estimates, the finding should be interpreted cautiously. Ancestry-specific subgroup findings were considered descriptive only and were not used for formal between-ancestry comparison because each subgroup contained fewer than three eligible estimates.

**Figure 6:**
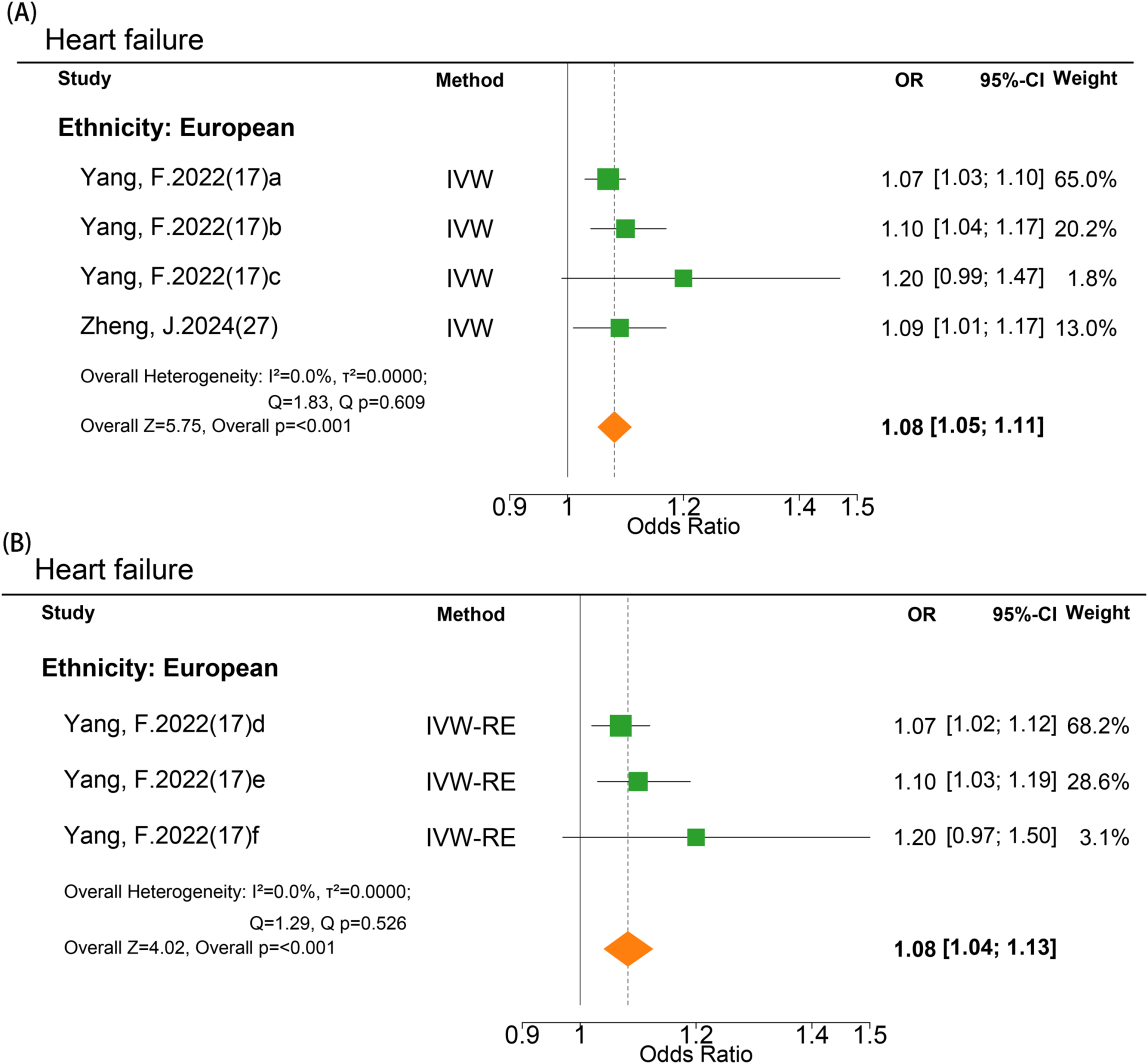
Meta-analysis of genetically predicted serum urate and heart failure. Forest plots showing the association between genetically predicted higher serum urate levels and heart failure. **(A)** shows the meta-analysis of primary IVW estimates, and **(B)** shows the meta-analysis of IVW-RE estimates. IVW and IVW-RE estimates were synthesized separately because they represented distinct primary analyses and were not combined in the same meta-analysis. Effect estimates are presented as odds ratios (ORs) with 95% confidence intervals (CIs), standardized to a 1.5 mg/dL increase in serum urate where applicable. Individual study estimates are shown as green squares, with square size proportional to the study weight; horizontal lines indicate 95% CIs. Orange diamonds represent pooled estimates. Estimates were synthesized using random-effects inverse-variance models. IVW, inverse-variance weighted method; IVW-RE, inverse-variance weighted random-effects method; OR, odds ratio; CI, confidence interval; I^2^, inconsistency statistic; τ^2^, between-study variance; Q, Cochran’s Q statistic.

For heart failure, IVW and IVW-RE estimates were synthesized separately because they represented distinct primary analyses in the original studies and were therefore not combined in a single meta-analysis. In the IVW analysis, 4 MR estimates retained after estimate-level screening were included. Genetically predicted higher serum urate was associated with a higher risk of heart failure (OR = 1.08, 95% CI: 1.05–1.11; I^2^ = 0.0%; τ^2^ = 0.0000; Q = 1.83, P = 0.609; **Figure 6A**). In the IVW-RE analysis, 3 MR estimates retained after estimate-level screening were included, and the pooled estimate showed a similar positive association (OR = 1.08, 95% CI: 1.04–1.13; I^2^ = 0.0%; τ^2^ = 0.0000; Q = 1.29, P = 0.526; **Figure 6B**).

### 3.5 Small-study effects and sensitivity analyses

Egger regression tests were performed for eligible overall meta-analyses and ancestry-restricted analyses with at least three MR estimates. All Egger test P values were greater than 0.05, providing no statistical evidence of funnel plot asymmetry or small-study effects. Therefore, trim-and-fill analyses were not performed. Given the limited number of estimates in several analyses, these Egger tests were interpreted as exploratory screening rather than definitive evidence against publication bias or small-study effects. The Egger regression test results are provided in **Supplementary Table 11**.

Leave-one-out sensitivity analyses were conducted by omitting one MR estimate at a time. The pooled associations for coronary heart disease, myocardial infarction, coronary artery disease, and heart failure remained directionally consistent after sequential omission of individual estimates, suggesting that the primary pooled estimates were not primarily driven by a single estimate. For atrial fibrillation, the direction of association was generally consistent, but the small number of eligible estimates limited interpretation. The leave-one-out analyses are shown in **Supplementary Figure 2**.

Hartung-Knapp adjusted random-effects analyses were further performed to evaluate the influence of small numbers of estimates on interval estimation and statistical inference. The overall associations for coronary heart disease, myocardial infarction, coronary artery disease, and heart failure remained statistically significant after Hartung-Knapp adjustment. In contrast, the atrial fibrillation association was no longer statistically significant after Hartung-Knapp adjustment. In ancestry-restricted analyses, the European subgroup findings for coronary artery disease and myocardial infarction remained statistically significant after Hartung-Knapp adjustment, whereas the European subgroup finding for coronary heart disease did not retain statistical significance. These results indicate that Hartung-Knapp adjustment provided additional sensitivity evidence for the overall coronary and heart failure analyses, but the atrial fibrillation and some ancestry-restricted findings should be interpreted cautiously. The Hartung-Knapp sensitivity analyses are summarized in **Supplementary Table 12**.

We additionally performed stepwise GWAS-source-restricted sensitivity analyses to further examine residual dependence among estimates sharing exposure or outcome GWAS sources. The selection of estimates for these analyses is summarized in **Supplementary Table 13**. After applying the stepwise exposure-first and then outcome-source restriction, three estimates remained for myocardial infarction and three estimates remained for coronary artery disease, allowing restricted meta-analysis. The restricted analysis for myocardial infarction remained positive (OR = 1.11, 95% CI: 1.03–1.19; I^2^ = 68.3%; τ^2^ = 0.0027; Q = 6.32, Q p = 0.042; **Supplementary Figure 3**). The restricted analysis for coronary artery disease also remained positive (OR = 1.19, 95% CI: 1.07–1.32; I^2^ = 65.6%; τ^2^ = 0.0060; Q = 5.81, Q p = 0.055; **Supplementary Figure 4**).

For coronary heart disease, only two estimates remained after combined GWAS-source restriction, and restricted meta-analysis was therefore not performed. Similarly, only one estimate remained for atrial fibrillation, two estimates remained for heart failure based on IVW estimates, and one estimate remained for heart failure based on IVW-RE estimates. These findings suggest that the associations for myocardial infarction and coronary artery disease were less sensitive to stricter GWAS-source restriction, whereas the evidence for coronary heart disease, atrial fibrillation, and heart failure was limited by the small number of estimates remaining after restriction and should therefore be interpreted more cautiously.

## 4 Discussion

In this systematic review and meta-analysis of MR studies, we synthesized available evidence on genetic susceptibility related to serum urate, hyperuricemia, and gout in relation to cardiovascular outcomes. After study-level reporting completeness assessment, estimate-level screening, exposure–outcome GWAS sample-overlap assessment, and MR-specific risk-of-bias evaluation, the final quantitative synthesis focused on genetically predicted serum urate and five cardiovascular outcomes. In the primary analyses, genetically predicted higher serum urate levels were positively associated with coronary heart disease, myocardial infarction, coronary artery disease, atrial fibrillation, and heart failure. However, after stepwise GWAS-source-restricted sensitivity analyses, only myocardial infarction and coronary artery disease retained at least three estimates and remained positively associated with genetically predicted serum urate. Therefore, the evidence was most consistent for myocardial infarction and coronary artery disease, whereas findings for coronary heart disease, atrial fibrillation, and heart failure should be interpreted more cautiously.

Among coronary outcomes, the most consistent evidence was observed for myocardial infarction and coronary artery disease. Although the primary analysis for coronary heart disease showed a positive association, fewer than three estimates remained after combined GWAS-source restriction, and this finding should therefore be interpreted with greater caution. In contrast, myocardial infarction and coronary artery disease remained positively associated with genetically predicted serum urate in the restricted sensitivity analyses, supporting a more stable coronary signal for these two outcomes. Leave-one-out analyses also suggested that the primary pooled coronary associations were not primarily driven by any single estimate. Nevertheless, the number of available estimates remained limited, and the coronary findings should be interpreted as evidence for selected coronary phenotypes rather than as evidence for a broad cardiovascular disease effect.

The interpretation of coronary outcomes requires attention to phenotype definitions. In the original MR studies and outcome GWAS datasets, coronary heart disease, coronary artery disease, and myocardial infarction were related but not identical outcomes. Myocardial infarction is a more specific acute event, commonly defined by acute myocardial injury in the setting of evidence of myocardial ischemia (50). In contrast, coronary artery disease and coronary heart disease may represent broader composite phenotypes, depending on the data source, and may include myocardial infarction, angina, chronic ischemic heart disease, coronary stenosis, or coronary revascularization (51). These differences were also evident in the extracted outcome definitions summarized in **Supplementary Table 7**. Therefore, we analyzed coronary heart disease, myocardial infarction, and coronary artery disease separately according to the labels and definitions used in the original MR studies. The directionally positive findings across these related outcomes may indicate a coronary signal, but direct comparison of effect sizes across coronary phenotypes should be made cautiously.

For heart failure, genetically predicted higher serum urate was associated with increased risk in both IVW and IVW-RE primary analyses. These two sets of estimates were synthesized separately because they represented distinct primary analyses in the original studies. The direction and magnitude of the associations were similar, no between-study heterogeneity was observed, and the overall association remained statistically significant after Hartung-Knapp adjustment. However, fewer than three estimates remained after combined GWAS-source restriction for both IVW and IVW-RE analyses. Therefore, the heart failure finding should be regarded as directionally consistent but limited by repeated or related GWAS-source contributions and by the small number of source-restricted estimates. In addition, heart failure outcome definitions differed across source datasets, including ICD-based definitions, clinical symptoms, elevated natriuretic peptides, imaging evidence, physician diagnosis, hospital admission records, or death records. Such variation may influence case ascertainment and should be considered when interpreting the pooled estimates.

For atrial fibrillation, the primary pooled analysis suggested a positive association with genetically predicted higher serum urate. However, only three eligible estimates were available, the association was no longer statistically significant after Hartung-Knapp adjustment, and only one estimate remained after combined GWAS-source restriction. Therefore, the atrial fibrillation result should be interpreted as suggestive rather than conclusive. Differences in atrial fibrillation ascertainment may also have contributed to uncertainty, because the source datasets used varying combinations of ICD codes, self-report, ECG records, surgical or procedural codes, and death records. Larger MR studies using independent exposure and outcome datasets with harmonized atrial fibrillation definitions are needed to clarify this association.

The present findings are partly consistent with previous observational and genetic evidence. Observational studies and umbrella reviews have reported associations between higher serum urate levels and multiple cardiovascular and metabolic outcomes, but such associations are difficult to separate from residual confounding by renal function, obesity, diet, diuretic use, insulin resistance, and other cardiometabolic factors (4). Previous MR studies have also produced mixed findings. Some studies did not support a clear causal effect of serum urate on coronary heart disease, ischemic stroke, or heart failure (8), whereas other evidence suggested that urate may be related to blood pressure or cardiovascular disease risk (10). By harmonizing effect scales, evaluating sample overlap, excluding estimates with high MR-specific risk of bias, and conducting GWAS-source-restricted sensitivity analyses, this study provides a focused synthesis of more comparable MR evidence. Differences across outcomes may reflect true biological heterogeneity, but may also arise from differences in GWAS sources, ancestry, instrument construction, outcome definitions, and MR methods.

Several biological mechanisms may explain why serum urate-related genetic susceptibility could be linked to selected cardiovascular outcomes. Experimental and clinical evidence suggests that urate may contribute to endothelial dysfunction, oxidative stress, vascular inflammation, activation of the renin-angiotensin system, vascular smooth muscle cell proliferation, and renal microvascular injury (2, 3, 52–54). These pathways may promote atherosclerosis, coronary plaque progression, myocardial injury, and cardiac remodeling. Such mechanisms are compatible with the observed associations for myocardial infarction and coronary artery disease and with the directionally positive heart failure findings. At the same time, urate has antioxidant properties in extracellular settings, and its biological effects may vary according to intracellular versus extracellular distribution, renal function, metabolic status, inflammatory state, and medication use. Therefore, serum urate is unlikely to have a uniform effect across all cardiovascular phenotypes.

The magnitude of the observed associations should also be interpreted cautiously. Although several pooled estimates were statistically significant, the effect sizes were modest, with ORs generally below 1.20 per 1.5 mg/dL genetically predicted increase in serum urate. Such estimates may be relevant at the population level if the exposure is common and sustained across the life course, but they have limited value for individual-level risk prediction. Therefore, these findings should not be interpreted as supporting universal serum urate screening or urate-lowering therapy for cardiovascular prevention. Any potential clinical application would need to consider baseline cardiovascular risk, gout status, renal function, treatment safety, cost-effectiveness, and evidence from randomized trials.

The discrepancy between MR evidence and randomized trial evidence should be interpreted carefully. MR estimates reflect the potential consequences of lifelong genetically influenced differences in serum urate levels, whereas randomized trials evaluate the effect of a specific urate-lowering intervention in a defined clinical population over a limited treatment period. The ALL-HEART trial found that allopurinol did not improve major cardiovascular outcomes compared with usual care in patients with ischemic heart disease but without gout (55). This negative trial result should not be dismissed simply as a matter of treatment timing. Several explanations may contribute to the discrepancy: genetic variants may influence urate levels and related biological pathways across the entire life course; drug therapy is usually initiated later in adulthood after atherosclerotic or myocardial disease has already developed; urate-related vascular or tissue injury, including possible crystal-associated inflammatory damage in susceptible individuals, may not be fully reversible by later urate lowering; and the pharmacological effect of allopurinol cannot be assumed to represent all genetically influenced urate pathways. Therefore, the present MR findings may support an etiological role of urate-related biology in selected cardiovascular outcomes, but they do not establish that urate-lowering therapy prevents cardiovascular events.

The sensitivity analyses provided additional context for interpreting the findings. Egger regression tests did not show statistical evidence of funnel plot asymmetry or small-study effects in eligible overall meta-analyses or ancestry subgroup analyses with at least three estimates; therefore, trim-and-fill analyses were not performed. Because the number of estimates was limited in several analyses, Egger test results should still be considered exploratory. Leave-one-out analyses suggested that the primary pooled associations for coronary heart disease, myocardial infarction, coronary artery disease, and heart failure were not primarily driven by any single estimate. Hartung-Knapp adjusted analyses provided additional sensitivity evidence for the overall associations with coronary heart disease, myocardial infarction, coronary artery disease, and heart failure, whereas the atrial fibrillation association did not remain statistically significant. In the stepwise GWAS-source-restricted sensitivity analyses, myocardial infarction and coronary artery disease remained positively associated with genetically predicted serum urate, but coronary heart disease, atrial fibrillation, and heart failure could not be re-pooled because fewer than three estimates remained. These findings indicate that the evidence was most consistent for myocardial infarction and coronary artery disease.

This study has several limitations. First, despite systematic screening and MR-specific risk-of-bias assessment, the number of eligible MR estimates was small for several outcomes, limiting the precision of pooled estimates and the power of Egger tests, subgroup analyses, Hartung-Knapp adjusted analyses, and GWAS-source-restricted sensitivity analyses. Stroke subtype and hypertension-related analyses could not be retained for final quantitative synthesis after estimate-level screening because fewer than three eligible estimates remained. In addition, binary hypertension outcomes were limited by heterogeneous case definitions and inconsistent reporting of antihypertensive medication handling across source GWAS datasets. Second, most available evidence came from European ancestry populations, and only limited evidence was available from Asian or other non-European populations. This restricts generalizability and limits reliable between-ancestry comparisons. Differences across ancestry-specific MR estimates should not be interpreted as direct evidence of biological differences between populations. They may reflect differences in allele frequencies, linkage disequilibrium structure, genetic instrument performance, environmental exposures, diet, lifestyle, outcome ascertainment, and GWAS statistical power. In particular, non-European exposure GWAS datasets were generally smaller, which may lead to less precise or more unstable MR estimates. Third, although exposure–outcome GWAS sample overlap was explicitly assessed and GWAS-source-restricted sensitivity analyses were added, complete participant-level overlap information was rarely available, and possible or unclear overlap could not be fully resolved. Fourth, residual horizontal pleiotropy, weak instrument bias, winner’s curse, and population stratification may still have affected some estimates. Fifth, outcome definitions and GWAS sources varied across original studies, particularly for coronary and heart failure outcomes, which may contribute to heterogeneity and limit direct comparability. Finally, the analysis was based on published aggregate MR estimates rather than individual participant data, limiting the ability to apply uniform instrument selection, harmonization, pleiotropy assessment, and multivariable adjustment across all outcomes.

In conclusion, genetically predicted higher serum urate levels were positively associated with selected coronary outcomes in the primary analyses, with the most consistent evidence observed for myocardial infarction and coronary artery disease after stepwise GWAS-source-restricted sensitivity analyses. Evidence for coronary heart disease, atrial fibrillation, and heart failure was more limited after stricter source restriction. These findings support a potential role of serum urate-related biology in selected cardiovascular outcomes, but they should not be interpreted as evidence for a broad causal effect on cardiovascular disease as a whole or as direct evidence that urate-lowering therapy prevents cardiovascular events.

## Data Availability

The original contributions presented in the study are included in the article/**Supplementary Material**. Further inquiries can be directed to the corresponding author.

## References

[1] Mensah GA Fuster V Murray CJL Roth GA Global Burden of Cardiovascular Diseases and Risks Collaborators . Global burden of cardiovascular diseases and risks, 1990-2022. J Am Coll Cardiol. (2023) 82:2350–473. doi: 10.1016/j.jacc.2023.11.007 38092509 PMC7615984

[2] Feig DI Kang DH Johnson RJ . Uric acid and cardiovascular risk. N Engl J Med. (2008) 359:1811–21. doi: 10.1056/NEJMra0800885 18946066 PMC2684330

[3] Johnson RJ Kang DH Feig D Kivlighn S Kanellis J Watanabe S . Is there a pathogenetic role for uric acid in hypertension and cardiovascular and renal disease? Hypertension (Dallas Tex 1979). (2003) 41:1183–90. doi: 10.1161/01.HYP.0000069700.62727.C5 12707287

[4] Li X Meng X Timofeeva M Tzoulaki I Tsilidis KK Ioannidis JP . Serum uric acid levels and multiple health outcomes: umbrella review of evidence from observational studies, randomised controlled trials, and mendelian randomisation studies. BMJ (Clinical Res Ed). (2017) 357:j2376. doi: 10.1136/bmj.j2376 28592419 PMC5461476

[5] Davies NM Holmes MV Davey Smith G . Reading mendelian randomisation studies: a guide, glossary, and checklist for clinicians. BMJ (Clinical Res Ed). (2018) 362:k601. doi: 10.1136/bmj.k601 30002074 PMC6041728

[6] Emdin CA Khera AV Kathiresan S . Mendelian randomization. JAMA. (2017) 318:1925–6. doi: 10.1001/jama.2017.17219 29164242

[7] Hemani G Zheng J Elsworth B Wade KH Haberland V Baird D . The MR-Base platform supports systematic causal inference across the human phenome. eLife. (2018) 7:e34408. doi: 10.7554/eLife.34408 29846171 PMC5976434

[8] Keenan T Zhao W Rasheed A Ho WK Malik R Felix JF . Causal assessment of serum urate levels in cardiometabolic diseases through a mendelian randomization study. J Am Coll Cardiol. (2016) 67:407–16. doi: 10.1016/j.jacc.2015.10.086 26821629 PMC5503188

[9] Testa A Prudente S Leonardis D Spoto B Sanguedolce MC Parlongo RM . A genetic marker of hyperuricemia predicts cardiovascular events in a meta-analysis of three cohort studies in high risk patients. Nutrition Metabolism Cardiovasc Dis NMCD. (2015) 25:1087–94. doi: 10.1016/j.numecd.2015.08.004 26607700

[10] Saito Y Tanaka A Node K Kobayashi Y . Uric acid and cardiovascular disease: a clinical review. J Cardiol. (2021) 78:51–7. doi: 10.1016/j.jjcc.2020.12.013 33388217

[11] Efstathiadou A Gill D McGrane F Quinn T Dawson J . Genetically determined uric acid and the risk of cardiovascular and neurovascular diseases: a mendelian randomization study of outcomes investigated in randomized trials. J Am Heart Assoc. (2019) 8:e012738. doi: 10.1161/JAHA.119.012738 31438759 PMC6755826

[12] Chen S Yang F Xu T Wang Y Zhang K Fu G . Genetically predicted serum uric acid levels and the risk of coronary artery disease in patients with diabetes: a mendelian randomization study. Nutrition Metabolism Cardiovasc Dis NMCD. (2021) 31:1832–9. doi: 10.1016/j.numecd.2021.03.007 33975736

[13] Gill D Cameron AC Burgess S Li X Doherty DJ Karhunen V . Urate, blood pressure, and cardiovascular disease: evidence from mendelian randomization and meta-analysis of clinical trials. Hypertension (Dallas Tex 1979). (2021) 77:383–92. doi: 10.1161/HYPERTENSIONAHA.120.16547 33356394 PMC7803439

[14] Lukkunaprasit T Rattanasiri S Ongphiphadhanakul B McKay GJ Attia J Thakkinstian A . Causal associations of urate with cardiovascular risk factors: two-sample mendelian randomization. Front Genet. (2021) 12:687279. doi: 10.3389/fgene.2021.687279 34306027 PMC8297413

[15] Lai B Yu HP Chang YJ Wang LC Chen CK Zhang W . Assessing the causal relationships between gout and hypertension: a bidirectional mendelian randomisation study with coarsened exposures. Arthritis Res Ther. (2022) 24:243. doi: 10.1186/s13075-022-02933-4 36309757 PMC9617405

[16] Wang K Shi X Zhu Z Hao X Chen L Cheng S . Mendelian randomization analysis of 37 clinical factors and coronary artery disease in east asian and european populations. Genome Med. (2022) 14:63. doi: 10.1186/s13073-022-01067-1 35698167 PMC9195360

[17] Yang F Hu T Cui H . Serum urate and heart failure: a bidirectional mendelian randomization study. Eur J Prev Cardiol. (2022) 29:1570–8. doi: 10.1093/eurjpc/zwac100 35578763

[18] Yang F Lu Y Chen S Wang K Hu T Cui H . Sex-specific effect of serum urate levels on coronary heart disease and myocardial infarction prevention: a mendelian randomization study. Nutrition Metabolism Cardiovasc Dis NMCD. (2022) 32:1266–74. doi: 10.1016/j.numecd.2022.01.022 35197211

[19] Zhu J Zeng Y Zhang H Qu Y Ying Z Sun Y . The association of hyperuricemia and gout with the risk of cardiovascular diseases: a cohort and mendelian randomization study in UK Biobank. Front Med. (2022) 8:817150. doi: 10.3389/fmed.2021.817150 35400029 PMC8985123

[20] Chen S Chen Z Xu Q Jiang X Lin C Ji J . Dual effects of serum urate on stroke risk and prognosis: insights from mendelian randomization. Front Neurol. (2024) 15:1359292. doi: 10.3389/fneur.2024.1359292 38628696 PMC11018999

[21] Hu F Yang J Wu S Yang C . Causal relationship between uric acid and stroke: a two-sample mendelian randomization study. Medicine. (2024) 103:e39591. doi: 10.1097/MD.0000000000039591 39432637 PMC11495787

[22] Chen X Cheng S Huang L Chen X Jin N Hong J . Serum uric acid, body mass index, and cardiovascular diseases: a multiple two-step mendelian randomization study. Nutrition Metabolism Cardiovasc Dis NMCD. (2024) 34:2386–94. doi: 10.1016/j.numecd.2024.05.023 39097442

[23] Deng T Liu X . Uric acid is associated with increased risk of myocardial infarction: results from NHANES 2009-2018 and bidirectional two-sample mendelian randomization analysis. Front Endocrinol. (2024) 15:1424070. doi: 10.3389/fendo.2024.1424070 39493770 PMC11527614

[24] Wang C Gao Y Smerin D Xiong X Chen Z Gu L . Genetically predicted type 2 diabetes mellitus mediates the causal association between plasma uric acid and ischemic stroke. Int Immunopharmacol. (2024) 134:112267. doi: 10.1016/j.intimp.2024.112267 38761781

[25] Xiong J Sun Y Huang H Liu Y Ling F Wei Y . The causal relationship between angina pectoris and gout based on two sample mendelian randomization. Pain Res Manage. (2024) 4564596. doi: 10.1155/2024/4564596 38633818 PMC11022507

[26] Zhang Y Lian Q Nie Y Zhao W . Causal relationship between serum uric acid and cardiovascular disease: a mendelian randomization study. Int J Cardiol Heart Vasculature. (2024) 54:101453. doi: 10.1016/j.ijcha.2024.101453 39411145 PMC11473680

[27] Zheng J Cen K Zhang J Zhang H Zhao M Hou X . Uric acid levels and heart failure: a mendelian randomization study. Nutrition Metabolism Cardiovasc Dis NMCD. (2024) 34:1008–13. doi: 10.1016/j.numecd.2023.12.023 38413359

[28] Zhu S Zhang M Cheng S Wang C Deng F . Assessing the causal associations of atrial fibrillation with serum uric acid level and gout: insights from a bidirectional mendelian randomization study. BMC Cardiovasc Disord. (2024) 24:638. doi: 10.1186/s12872-024-04319-7 39538126 PMC11562312

[29] Li D Lin J Yang H Zhou L Li Y Xu Z . Causal association of modifiable factors with cardiometabolic multimorbidity: an exposome-wide mendelian randomization investigation. Cardiovasc Diabetol. (2025) 24:241. doi: 10.1186/s12933-025-02790-w 40481456 PMC12143095

[30] Li J Li Y Zhang S Shi H . A mendelian randomization study of serum uric acid in metabolic and cardiovascular risk in east asian populations. Medicine. (2025) 104:e44135. doi: 10.1097/MD.0000000000044135 40922357 PMC12419271

[31] Wang S Mei S Ma X Wuyun Q Zhou L Luo Q . Causal relationship between serum uric acid and atherosclerotic disease: a mendelian randomization and transcriptomic analysis. Biomedicines. (2025) 13:1838. doi: 10.3390/biomedicines13081838 40868093 PMC12383975

[32] Xu J Zhao J Gu J Wang W Chen J . Serum uric acid levels as a causal factor in hypertension: insights from mendelian randomization analysis. Clin Exp Hypertension (New York NY 1993). (2025) 47:2496514. doi: 10.1080/10641963.2025.2496514 40325623

[33] Hou X Hou X Cen K Zhu Y Zhu Z Zhang Z . Uric acid levels and cardiovascular and cerebrovascular diseases: a mendelian randomization study. Cerebrovascular Dis (Bsl Switz). (2025) 54:795–803. doi: 10.1159/000541624 39369690

[34] Yu S Wang W Gao Y Zhou J Chu Y . Serum urate and atrial fibrillation: a bidirectional mendelian randomization study. Clin Cardiol. (2025) 48:e70089. doi: 10.1002/clc.70089 39871634 PMC11772717

[35] Zhang X Wang X Zhang W Li M Li Q . Insights into modifiable risk factors of atrial fibrillation: a comprehensive mendelian randomization study. Postgrad Med J. (2026) 102:147–57. doi: 10.1093/postmj/qgaf141 40886111

[36] Li X Meng X Spiliopoulou A Timofeeva M Wei WQ Gifford A . MR-PheWAS: exploring the causal effect of SUA level on multiple disease outcomes by using genetic instruments in UK Biobank. Ann Rheumatic Dis. (2018) 77:1039–47. doi: 10.1136/annrheumdis-2017-212534 29437585 PMC6029646

[37] Chiang KM Tsay YC Vincent Ng TC Yang HC Huang YT Chen CH . Is hyperuricemia, an early-onset metabolic disorder, causally associated with cardiovascular disease events in Han Chinese? J Clin Med. (2019) 8:1202. doi: 10.3390/jcm8081202 31408958 PMC6723695

[38] Hong M Park JW Yang PS Hwang I Kim TH Yu HT . A mendelian randomization analysis: the causal association between serum uric acid and atrial fibrillation. Eur J Clin Invest. (2020) 50:e13300. doi: 10.1111/eci.13300 32474920

[39] He Y Feng J Zhang B Wu Q Zhou Y He D . Serum uric acid levels and risk of cardiovascular disease in type 2 diabetes: results from a cross-sectional study and mendelian randomization analysis. Front Endocrinol. (2023) 14:1251451. doi: 10.3389/fendo.2023.1251451 38027101 PMC10664243

[40] Li X Meng X He Y Spiliopoulou A Timofeeva M Wei WQ . Genetically determined serum urate levels and cardiovascular and other diseases in UK Biobank cohort: a phenome-wide mendelian randomization study. PloS Med. (2019) 16:e1002937. doi: 10.1371/journal.pmed.1002937 31626644 PMC6799886

[41] Palmer TM Nordestgaard BG Benn M Tybjærg-Hansen A Davey Smith G Lawlor DA . Association of plasma uric acid with ischaemic heart disease and blood pressure: mendelian randomisation analysis of two large cohorts. BMJ (Clinical Res Ed). (2013) 347:f4262. doi: 10.1136/bmj.f4262 23869090 PMC3715134

[42] White J Sofat R Hemani G Shah T Engmann J Dale C . Plasma urate concentration and risk of coronary heart disease: a mendelian randomisation analysis. Lancet Diabetes Endocrinol. (2016) 4:327–36. doi: 10.1016/S2213-8587(15)00386-1 26781229 PMC4805857

[43] Köttgen A Albrecht E Teumer A Vitart V Krumsiek J Hundertmark C . Genome-wide association analyses identify 18 new loci associated with serum urate concentrations. Nat Genet. (2013) 45:145–54. doi: 10.1038/ng.2500 23263486 PMC3663712

[44] Fall T Gustafsson S Orho-Melander M Ingelsson E . Genome-wide association study of coronary artery disease among individuals with diabetes: the UK Biobank. Diabetologia. (2018) 61:2174–9. doi: 10.1007/s00125-018-4686-z 30003307 PMC6133153

[45] Tin A Marten J Halperin Kuhns VL Li Y Wuttke M Kirsten H . Target genes, variants, tissues and transcriptional pathways influencing human serum urate levels. Nat Genet. (2019) 51:1459–74. doi: 10.1038/s41588-019-0504-x 31578528 PMC6858555

[46] Sakaue S Kanai M Tanigawa Y Karjalainen J Kurki M Koshiba S . A cross-population atlas of genetic associations for 220 human phenotypes. Nat Genet. (2021) 53:1415–24. doi: 10.1038/s41588-021-00931-x 34594039 PMC12208603

[47] Aragam KG Jiang T Goel A Kanoni S Wolford BN Atri DS . Discovery and systematic characterization of risk variants and genes for coronary artery disease in over a million participants. Nat Genet. (2022) 54:1803–15. doi: 10.1038/s41588-022-01233-6 36474045 PMC9729111

[48] Roselli C Chaffin MD Weng LC Aeschbacher S Ahlberg G Albert CM . Multi-ethnic genome-wide association study for atrial fibrillation. Nat Genet. (2018) 50:1225–33. doi: 10.1038/s41588-018-0133-9 29892015 PMC6136836

[49] Nielsen JB Thorolfsdottir RB Fritsche LG Zhou W Skov MW Graham SE . Biobank-driven genomic discovery yields new insight into atrial fibrillation biology. Nat Genet. (2018) 50:1234–9. doi: 10.1038/s41588-018-0171-3 30061737 PMC6530775

[50] Thygesen K Alpert JS Jaffe AS Chaitman BR Bax JJ Morrow DA . Fourth universal definition of myocardial infarction (2018). Circulation. (2018) 138:e618–51. doi: 10.1161/CIR.0000000000000617 30571511

[51] Nelson CP Goel A Butterworth AS Kanoni S Webb TR Marouli E . Association analyses based on false discovery rate implicate new loci for coronary artery disease. Nat Genet. (2017) 49:1385–91. doi: 10.1038/ng.3913 28714975

[52] Mazzali M Hughes J Kim YG Jefferson JA Kang DH Gordon KL . Elevated uric acid increases blood pressure in the rat by a novel crystal-independent mechanism. Hypertension (Dallas Tex 1979). (2001) 38:1101–6. doi: 10.1161/hy1101.092839 11711505

[53] Kanellis J Watanabe S Li JH Kang DH Li P Nakagawa T . Uric acid stimulates monocyte chemoattractant protein-1 production in vascular smooth muscle cells via mitogen-activated protein kinase and cyclooxygenase-2. Hypertension (Dallas Tex 1979). (2003) 41:1287–93. doi: 10.1161/01.HYP.0000072820.07472.3B 12743010

[54] Yu MA Sánchez-Lozada LG Johnson RJ Kang DH . Oxidative stress with an activation of the renin-angiotensin system in human vascular endothelial cells as a novel mechanism of uric acid-induced endothelial dysfunction. J Hypertens. (2010) 28:1234–42. doi: 10.1097/HJH.0b013e328337da1d 20486275

[55] Mackenzie IS Hawkey CJ Ford I Greenlaw N Pigazzani F Rogers A . Allopurinol versus usual care in UK patients with ischaemic heart disease (ALL-HEART): a multicentre, prospective, randomised, open-label, blinded-endpoint trial. Lancet (Lond Engl). (2022) 400:1195–205. doi: 10.1016/S0140-6736(22)01657-9 36216006

