# Supplementary material for: Genetic susceptibility to uric acid and selected cardiovascular outcomes: a systematic review and meta-analysis of Mendelian randomization studies
Source: Front Endocrinol (Lausanne). 2026 Jul 24;17:1880061. doi: 10.3389/fendo.2026.1880061 (PMC13447163; doi:10.3389/fendo.2026.1880061)
Supplement: Supplementary file 1 [file DataSheet2.pdf]

Supplementary Figure 1. European ancestry-restricted meta-analysis for genetically predicted serum urate and coronary artery disease

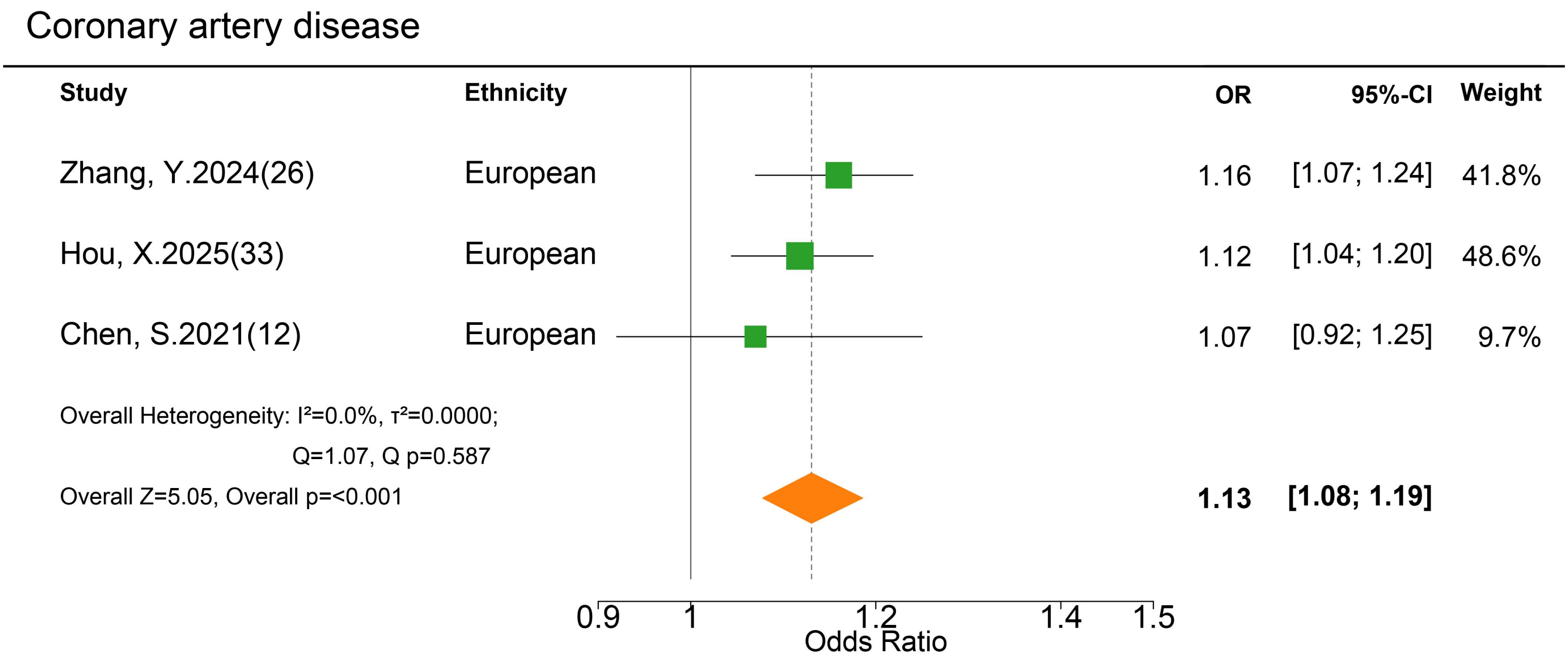

Forest plot showing the random-effects meta-analysis restricted to European ancestry estimates for the association between genetically predicted serum urate and coronary artery disease. Three European ancestry MR estimates were included. The pooled estimate was calculated using an inverse-variance random-effects model on the log(OR) scale with REML estimation of between-study variance. Green squares represent individual MR estimates, with size proportional to random-effects weight; horizontal lines represent 95% confidence intervals; the orange diamond represents the pooled estimate. The solid vertical line indicates the null value of OR = 1, and the dashed vertical line indicates the pooled estimate. This ancestry-restricted analysis was presented descriptively and was not used for formal between-ancestry comparison. MR, Mendelian randomization; OR, odds ratio; CI, confidence interval; REML, restricted maximum likelihood.

Supplementary Figure 2. Leave-one-out sensitivity analyses for eligible meta-analyses.

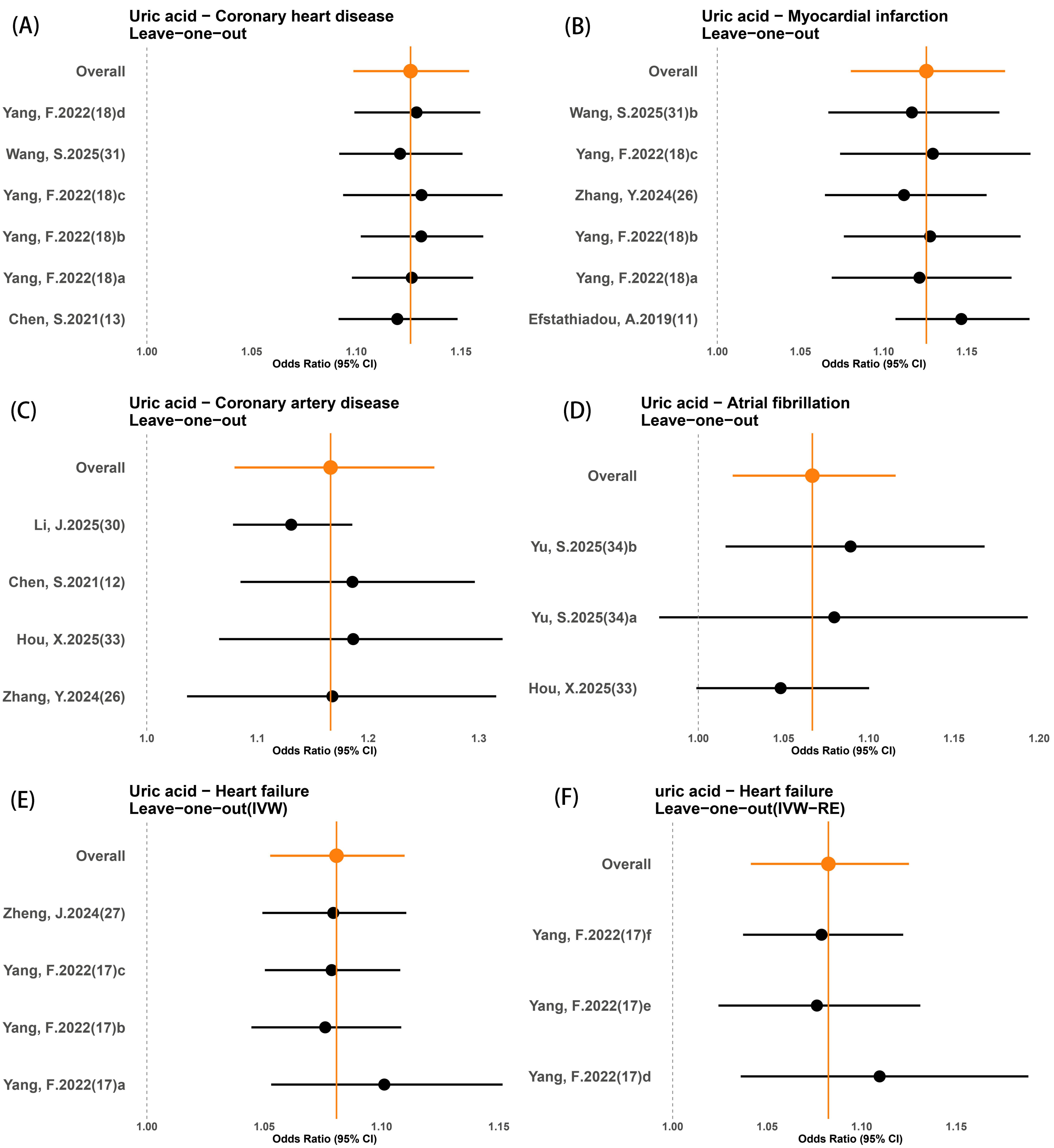

Leave-one-out sensitivity analyses were performed by sequentially omitting one MR estimate at a time and recalculating the pooled estimate. Panels show analyses for coronary heart disease, myocardial infarction, coronary artery disease, atrial fibrillation, heart failure based on IVW estimates, and heart failure based on IVW-RE estimates. Black points and horizontal lines represent recalculated pooled odds ratios (ORs) and 95% confidence intervals (CIs) after omission of the corresponding estimate. Orange points and vertical orange lines indicate the overall pooled estimates from the primary meta-analyses. The grey dashed vertical line indicates the null value of OR = 1. These analyses were used to evaluate whether the pooled associations were driven by any single MR estimate. MR, Mendelian randomization; OR, odds ratio; CI, confidence interval; IVW, inverse-variance weighted method; IVW-RE, inverse-variance weighted random-effects method.

Supplementary Figure 3. Stepwise GWAS-source-restricted sensitivity analysis for genetically predicted serum urate and myocardial infarction

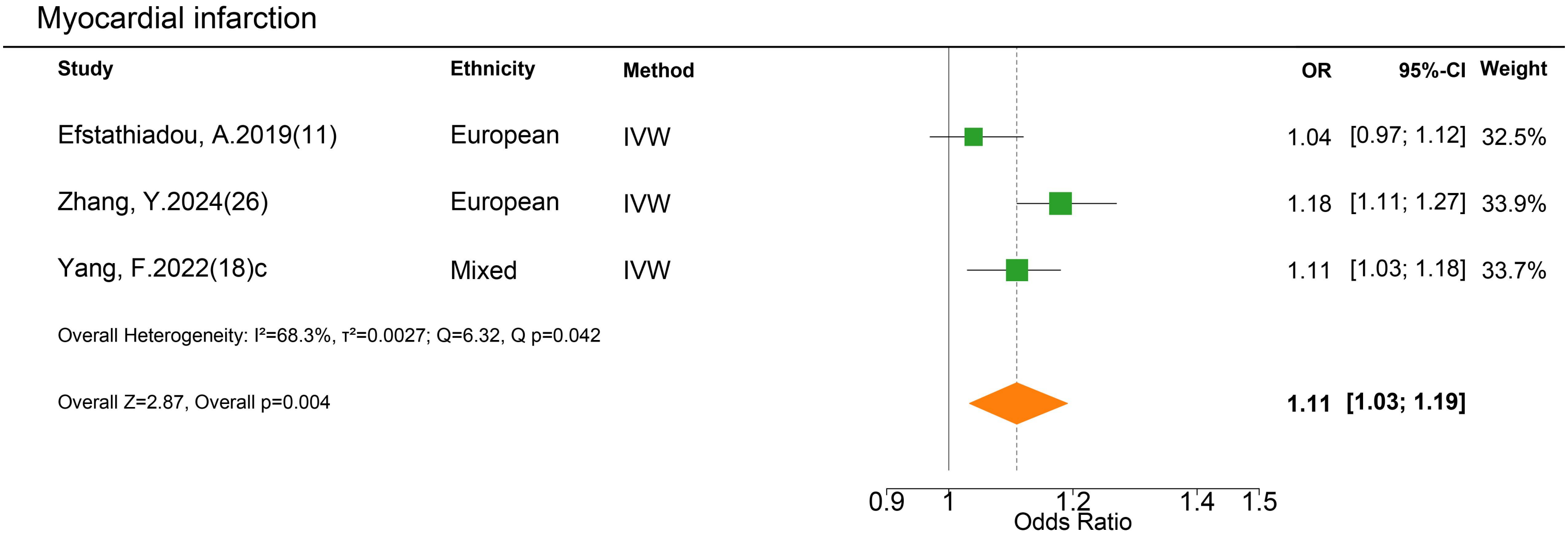

Forest plot showing the restricted random-effects meta-analysis of genetically predicted serum urate and myocardial infarction after stepwise GWAS-source restriction. Estimates were selected by first retaining only one estimate from the same or closely related exposure GWAS source and then retaining only one estimate from the same or closely related outcome GWAS source. Three estimates remained after restriction and were included in the meta-analysis. The pooled estimate was calculated using a random-effects inverse-variance model on the log(OR) scale with REML estimation of between-study variance. Squares represent individual MR estimates, with size proportional to random-effects weight; horizontal lines represent 95% confidence intervals; the orange diamond represents the pooled estimate. IVW, inverse-variance weighted; OR, odds ratio; CI, confidence interval; GWAS, genome-wide association study; REML, restricted maximum likelihood.

Supplementary Figure 4. Stepwise GWAS-source-restricted sensitivity analysis for genetically predicted serum urate and coronary artery disease

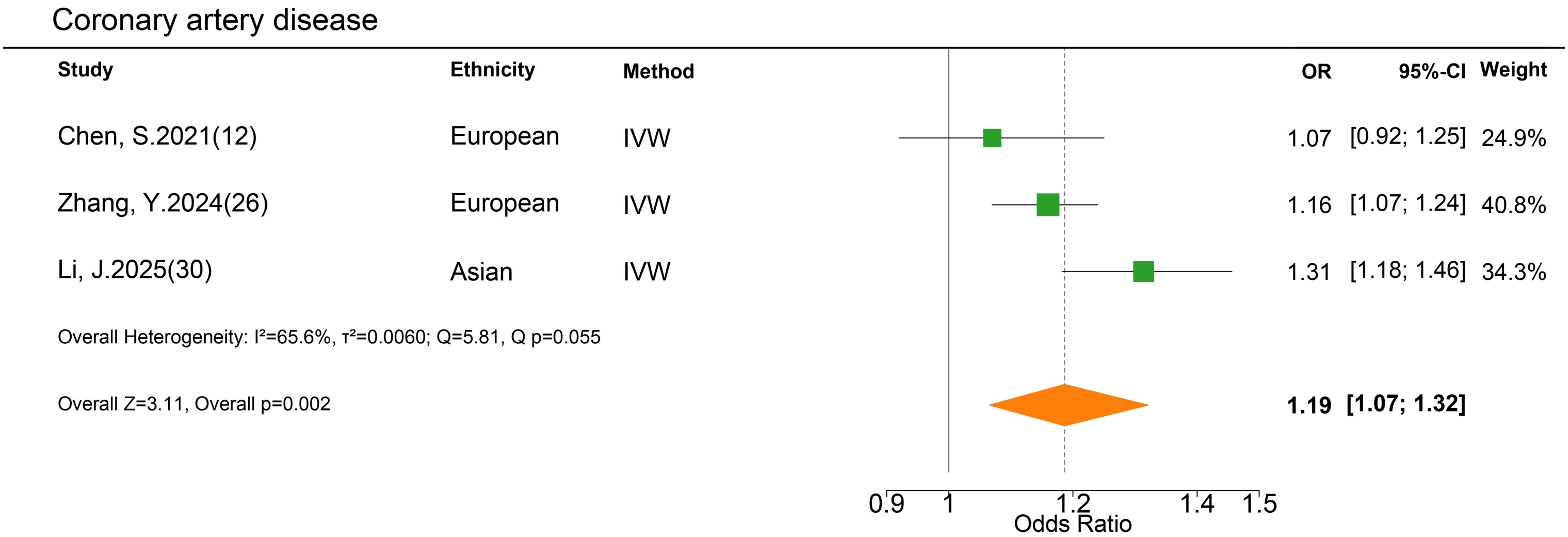

Forest plot showing the restricted random-effects meta-analysis of genetically predicted serum urate and coronary artery disease after stepwise GWAS-source restriction. Estimates were selected by first retaining only one estimate from the same or closely related exposure GWAS source and then retaining only one estimate from the same or closely related outcome GWAS source. Three estimates remained after restriction and were included in the meta-analysis. The pooled estimate was calculated using a random-effects inverse-variance model on the log(OR) scale with REML estimation of between-study variance. Squares represent individual MR estimates, with size proportional to random-effects weight; horizontal lines represent 95% confidence intervals; the orange diamond represents the pooled estimate. IVW, inverse-variance weighted; OR, odds ratio; CI, confidence interval; GWAS, genome-wide association study; REML, restricted maximum likelihood.
